# Multifaceted Role of Matrix Metalloproteinases in Neurodegenerative Diseases: Pathophysiological and Therapeutic Perspectives

**DOI:** 10.3390/ijms22031413

**Published:** 2021-01-30

**Authors:** Tapan Behl, Gagandeep Kaur, Aayush Sehgal, Shaveta Bhardwaj, Sukhbir Singh, Camelia Buhas, Claudia Judea-Pusta, Diana Uivarosan, Mihai Alexandru Munteanu, Simona Bungau

**Affiliations:** 1 Department of Pharmacology, Chitkara College of Pharmacy, Chitkara University, Chandigarh 140401, Punjab, India; kgagandeep060@gmail.com (G.K.); aayushsehgal00@gmail.com (A.S.); singh.sukhbir12@gmail.com (S.S.); 2 Department of Pharmacology, GHG Khalsa College of Pharmacy, Gurusar Sadhar, Ludhiana 141104, Punjab, India; sshwetasharma1987@gmail.com; 3 Department of Morphological Disciplines, Faculty of Medicine and Pharmacy, University of Oradea, 410073 Oradea, Romania; cameliabuhas@yahoo.com (C.B.); claupustaml@yahoo.com (C.J.-P.); 4 Department of Preclinical Disciplines, Faculty of Medicine and Pharmacy, University of Oradea, 410073 Oradea, Romania; diana.uivarosan@gmail.com; 5 Department of Medical Disciplines, Faculty of Medicine and Pharmacy, University of Oradea, 410073 Oradea, Romania; mihaimunteanual@yahoo.com; 6 Department of Pharmacy, Faculty of Medicine and Pharmacy, University of Oradea, 410028 Oradea, Romania

**Keywords:** neurodegenerative diseases, matrix metalloproteases, Alzheimer’s disease, multiple sclerosis, Parkinson’s disease

## Abstract

Neurodegeneration is the pathological condition, in which the nervous system or neuron loses its structure, function, or both, leading to progressive degeneration or the death of neurons, and well-defined associations of tissue system, resulting in clinical manifestations. Neuroinflammation has been shown to precede neurodegeneration in several neurodegenerative diseases (NDs). No drug is yet known to delay or treat neurodegeneration. Although the etiology and potential causes of NDs remain widely indefinable, matrix metalloproteinases (MMPs) evidently have a crucial role in the progression of NDs. MMPs, a protein family of zinc (Zn^2+^)-containing endopeptidases, are pivotal agents that are involved in various biological and pathological processes in the central nervous system (CNS). The current review delineates the several emerging evidence demonstrating the effects of MMPs in the progression of NDs, wherein they regulate several processes, such as (neuro)inflammation, microglial activation, amyloid peptide degradation, blood brain barrier (BBB) disruption, dopaminergic apoptosis, and α-synuclein modulation, leading to neurotoxicity and neuron death. Published papers to date were searched via PubMed, MEDLINE, etc., while using selective keywords highlighted in our manuscript. We also aim to shed a light on pathophysiological effect of MMPs in the CNS and focus our attention on its detrimental and beneficial effects in NDs, with a special focus on Parkinson’s disease (PD), amyotrophic lateral sclerosis (ALS), Alzheimer’s disease (AD), multiple sclerosis (MS), and Huntington’s disease (HD), and discussed various therapeutic strategies targeting MMPs, which could serve as potential modulators in NDs. Over time, several agents have been developed in order to overcome challenges and open up the possibilities for making selective modulators of MMPs to decipher the multifaceted functions of MMPs in NDs. There is still a greater need to explore them in clinics.

## 1. Introduction

Metalloproteinases (MPs), an important protease family, including matrix metalloproteinases (MMPs), are vital for several physiological and pathological processes, which have been obtained in the past two decades [1,2]. In the central nervous system (CNS), MPs act via the regulation of signaling cascade during synaptic dysfunction, blood-brain barrier (BBB) disruption, neuroinflammation, or neuronal loss [3,4]. In CNS, the MMPs are present in different cells, among which some of the family members are implicated in the development, repair, and injury in neurodegenerative diseases (NDs), which makes them attractive for therapeutic targets in certain diseases [2,5]. MMPs are pivotal for the development of brain due to their correlation with essential neurophysiological processes and functions [6,7]. In certain pathological conditions, such as neuroinflammatory conditions and neurodegenerative disorders (NDs) including Parkinson’s disease (PD), amyotrophic lateral sclerosis (ALS), Alzheimer’s disease (AD), multiple sclerosis (MS), and Huntington’s disease (HD), augmented MMP expression has been recognized, resulting in the exacerbation of neuroinflammation-induced brain damage [8].

NDs are a debilitating group of disorders that involves considerable neuronal deterioration in certain regions of the brain and well-defined associations of tissue system, resulting in clinical manifestations [9,10]. The activities of MMPs are stringently controlled and its deregulation leads to certain pathologies in NDs. MMPs are widely involved in the development of neurons, having the capability to alter the response to NDs [5]. In various NDs, neuroinflammation has been shown to precede neurodegeneration, wherein MMPs are vital in neuroinflammation and perhaps implicated in neurodegeneration [8]. MMPs regulates several processes, including inflammation, microglial activation, blood brain barrier (BBB) disruption, dopaminergic (DAergic) apoptosis, and α-synuclein modulation [3,11,12]. At CNS barriers, the activity of MMP prompts the rise in permeability by changing the extracellular matrix (ECM) and tight junctional properties [13].

On the other hand, the activation of MMPs is known to take part in angiogenesis, neurogenesis, and tissue repair [1]. MMPs are pivotal in the pathological conditions in the brain as a part of neuroinflammatory response in ischemic injury, infection, and vascular dementias causes [5]. MMPs are divided into four primary categories, as follows: Stromelysins, Collagenases, Gelatinases, and Film sort (MT)-MMPs. MMP-2 and -9 belong to Gelatinases, are part of neurogenesis and angiogenesis through basal lamina corruption, thus resulting in cell death. MT-MMPs activate development components and proteases at the cell surface. Stromelysins (MMP-11, -10, -7, and -3) are known to degrade the ECM [14,15,16,17]. In addition, studies have also indicated MMPs in the vascular cognitive impairment (VCI)-associated neurodegeneration [5,18]. They have been subject to wide research, due to the leading role of MMPs’ in neuroinflammation and several NDs [19].

The present review is an overview that is related to the multifaceted role of MMPs in NDs via cellular functions (such as remodeling and degradation of the ECM and proteolysis of cell signaling factors). We aimed to shed a light on pathophysiological activities of MMPs in the CNS, with our attention being focused on its detrimental/beneficial effects in NDs, with special approach on PD, AD, ALS, MS, and HD. Moreover, various therapeutic strategies targeting MMPs, which could serve novel treatment for NDs, were discussed.

## 2. An Overview of Matrix Metalloproteinases (MMPs)-Basic Structure and Function

Belonging to a superfamily of metzincin, like the reprolysins, serralysins, astacins, and adamalysins or disintegrin metalloproteinases (ADAMs), MMPs form calcium (Ca^2+^)- and zinc (Zn^2+^)-dependent endopeptidases involved in the regulation of biological functions and several pathological processes, once activated [20,21,22]. MMPs have the ability to digest diverse forms of substrates, including the ECM components and basement membrane [22,23]. MPs play a vital role in ECM remodeling by cell surface protein activation, the proteolytic degradation of ECM components, and shedding of membrane-bound receptor molecules. They are widely known for the regulation of activity of chemokines, growth factors, cell receptors, and other proteinases, and it regulates certain biological processes, such as cell differentiation, survival, migration, and proliferation, in different forms of cellular processes and functions [23,24].

The basic structure of MPs consists of a catalytic domain and pro peptide sequence [25,26]. In addition, MMPs comprise multiple domains, as follows: pro peptide-maintains MMPs’ latency; N-terminal signal peptide-cleaved in the secretory pathway; catalytic domain-holds the Zn^2+^ ion that is required for the enzymatic activity; hinge region–serve as linking sequences; and, C-terminal hemo-pexin-like (PEX) domain—as needed for the identification of substrate [27]. Alongside common domains as mentioned, some of the MMPs have additional domains and different peptide structures [28]. MMPs are divided into four primary categories: Stromelysins (MMP-3, -7, -10, and -11), Collagenases (MMP-1, -8, and -13), Gelatinases (MMP-2 and -9), and Membrane type (MT)-MMPs [14,15,16,17]. The synthesis of all these MMPs occurs with a common N-terminal sequence, which is further cleaved as proenzyme in the endoplasmic reticulum [29,30]. MT-MMPs, at the C-terminals, contain the glycosyl phosphatidyl inositol (GPI)-anchored domain, which is anchored to the cell membrane via formation of covalent bonds [31]. The production of MMPs occurs as pro-MMP (zymogens) via cysteine switch mechanism. Pro-MMP is activated by free radicals or other enzymes, wherein the binding of the thiol group (present in N-terminal domain) to the Zn^2+^ atom blocks its active site; removal or blockade of thiol group initiates the activation of MMPs [32]. MMPs are known to be crucial agents in several pathological processes and as biological regulators in the body, once activated. In addition, MMPs aid in the degradation of ECM and its components along with several non-matrix substances [33,34].

ECM is a vital structure that provides an adhesion site for different cells and aids several physiological processes. It also acts as a storage region for multiple growth factors, proteins, and signaling molecules, thereby affecting cell migration and development. ECM mainly contains proteoglycans, glycosaminoglycans, and fibrous proteins (laminin, fibronectin, and collagen). ECM proteolysis or cleavage by MMP influences embryogenesis, cell migration, and various processes in adult organism and during development [35,36]. Being widely known to play a vital role in cell adhesion and intracellular signaling, ADAMs (MP and a-disintegrin) are transmembrane-anchored MPs that have identical catalytic MMP domains, but do not have a PEX domain, and rather have three extra epidermal growths factor-like domains together with disinterring domain. At the C-terminal region, ADAMTS (MP with thrombospondin motifs and a-disintegrin) family members comprise different type-1 thrombospondin (TSP-1) domains [23,24,37]. The tissue inhibitors of metalloproteinases (TIMPs) are 21–28 kDa proteins that bind the MMPs’ active site in 1:1 [28,38]. The tight regulation of MMP activity occurs via pro-MMP proteolytic activation and its natural inhibitor, TIMPs. Insufficient TIMP control and MPs overexpression leads to the dysregulation of tissue remodeling, which results in various diseases, including NDs [3,39,40,41]. A variety of stimuli activates MMPs, including several growth factors and proinflammatory cytokines, which can commence an intracellular signaling process, resulting in the activation of nuclear factor kappa light chain enhancer of activated B cells (NFkB), Activator protein -1 (AP-1), or E26 transformation-specific (ETS) transcription factors, with the subsequent transcription of MMP [42]. Moreover, high reactive oxygen species (ROS) levels can induce and initiate MMPs activation [43]. In spite of the fact that early attempts of targeting MMPs were ineffective in clinical trials, MPs still persist to be potential therapeutic target, depending on their vital role in the disease progression [26].

## 3. Involvement of MMPs in CNS

**Figure 1:**
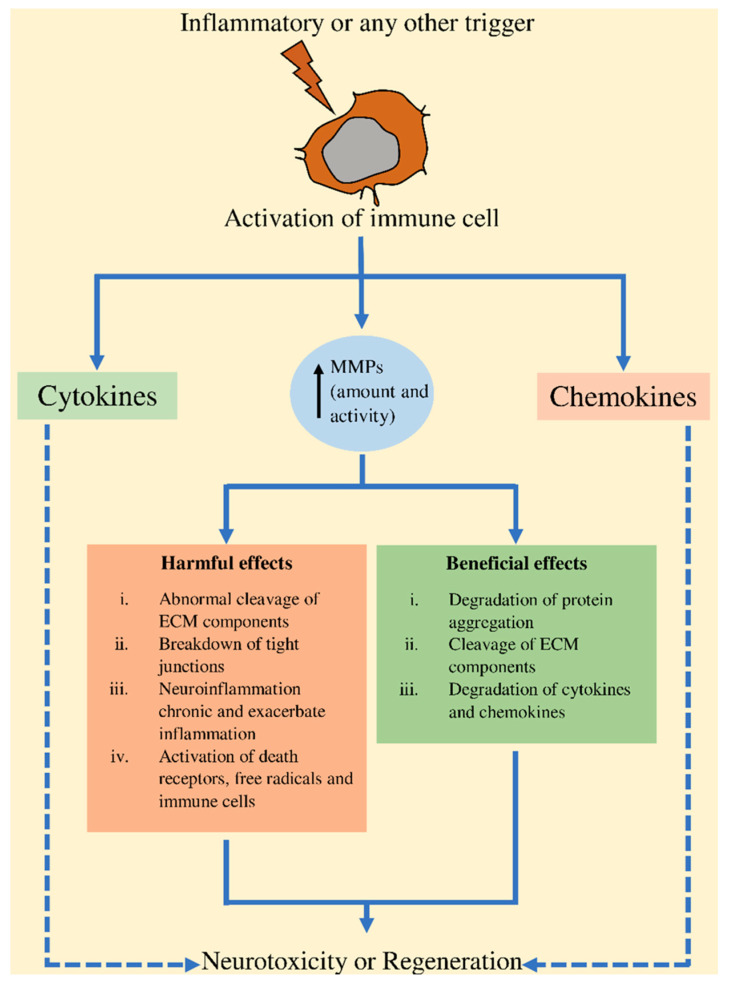
Representation of MMP activation, their interactions with chemokines and cytokines, and the outcome. ECM, extracellular matrix; MMPs, matrix metalloproteinases.

MMPs have been observed in the CNS, which are produced by endothelial cells, microglia, oligodendrocytes, neurons, and astrocytes [44]. Under normal conditions, it has been observed that MMPs are either present or absent at undetectable concentrations and expressed at modest levels in the mature brain. A dysregulation in the MMP activity could alter the balance, inducing a continuation of the inflammation [45,46]. Furthermore, MMPs are engaged in the maintenance of CNS barrier in order to enhance the barrier permeability during inflammation. The suggested process involves ECM components’ degradation (e.g., collagen and laminin), which mainly impede access to different substances through barriers and assist the cellular structures [47,48,49]. In addition, the augmented activity of MMP negatively affects the function of tight junctions at the brain barriers [49,50,51,52]. Instead, some MMPs have been observed to activate free radicals and proinflammatory cytokines that augments the inflammation, inducing BBB disruption [4,13,15]. Upon inflammation conditions, MMPs are secreted, thereby contributing to disrupting the barriers and aggravating the inflammation [53,54]. Together with TIMPs, MMPs contribute to physiology of nervous system during neurogenesis, ontogenesis, neuronal plasticity, and angiogenesis [21,55]. In neuronal plasticity, MMPs involvement has been linked to influence memory and learning ability and underlying long-term potentiation (LTP) [22,56,57]. Figure 1 presents the representation of MMP activation, their interactions with chemokines and cytokines, and the outcome.

Furthermore, MMPs have been involved in the migration of neural cells, synaptogenesis, and regeneration of nervous tissue [6,58,59]. MMP upregulation has been widely recognized in an innumerable pathological condition, including neuronal death, hypoxia/ischemia, neuroinflammation, BBB disruption, as well as demyelination [6,60,61,62,63]. Neuroinflammation is a common characteristic of such CNS pathologies, wherein the activation and production of particular MMPs initiates or amplifies by immune cells (lymphocytes, macrophages, neutrophils, etc.) or neural cells (microglia, astrocytes, endothelial cells, etc.) [64]. Nevertheless, being considered to be multifaceted enzymes, MMPs mediate various biological and pathological pathways in CNS, where the outcome needs to be carefully assessed while allowing their inhibition, so as to anticipate/avoid undesirable side effects [65].

### The Links between MMPs and Aquaporin-4

This review focuses on an important point, which is the role of MMPs, with a particular focus on BBB disruption. Lines of evidence showed that neuroinflammation and pro-inflammatory cytokines secretion caused aquaporin-4 (AQP, a water channel protein, encoded by the AQP4 gene) disorganized, leading to brain edema. This has been recently demonstrated by Kitchen et al., where they showed that the targeting AQP4 following ischemia and hypoxia not only reduces edema, sbut also stabilizes the BBB/BSCB barriers [66]. Multiple aspects of the links between MMPs and AQPs (in particular AQP4) have been established by recent of previous published studies [67,68,69]. The results of the study by Higashida et al. showed that AQP-4 plays a role in the formation of brain edema and BBB disruption via a molecular pathway cascade involving MMP-9 and AQP4. The pharmacological blockade of this pathway may provide a novel therapeutic strategy [67]. The study conducted by Cao et al. also showed that hydrogen sulphide attenuated brain edema formation reduces the MMP-9 expression and suppresses AQP4 expression via the alleviation of glia activation and pro-inflammatory cytokines secretion [68]. A study by Li et al. showed that the expressions of MMP-9 and AQP4 were increased in the vehicle group that was associated with cerebral vasogenic edema or cytotoxic edema. The MMP-9 and AQP4 up-regulations were significantly inhibited by the administration of astragaloside IV, proposing that the anti-edema potential of astragaloside IV was related to the regulation of MMP-9 and AQP4 [69].

AQPs are historically known to be passive transporters of water. Evidence in the last decade has highlighted the diverse function of AQPs beyond water homeostasis [70]. Additionally, a subgroup of AQP water channels also facilitates transmembrane diffusion of small, polar solutes, not only water, aquaglyceroporin [71,72]. The increased AQP4 expression and redistribution/surface localization can be two different concepts. Previous studies have shown an increased in AQP4 membrane localization in primary human astrocytes that was not accompanied by a change in AQP4 protein expression levels [73,74]

Moreover, AQPs have been validated as an important drug target, but there is no single drug that has yet been approved to successfully target it, as there are not many studies that investigated the mentioned future therapies in term of the communication between MMPs and AQPs (mainly AQP4) [75,76].

Brain injury and ischemia are known to reduce blood supply and, hence, oxygen (hypoxia), which affects the energy homeostasis in the brain and BBB remodeling. It was highlighted the role of the changes in brain energy metabolism and how ischemia/hypoxia affects different signaling pathways that are known to also affect the adhesion to endothelial cells and, hence, transport through BBB and toxicity to the brain [8,77,78,79]. It has been illustrated that the brain has a high energy requirement due to the high number of neurons and maintenance of a delicate interplay between neurotransmission, energy metabolism, and plasticity. Energy balance disturbances, to quality control of mitochondria or to glia-neuron metabolic interaction, may result in malfunctioning of brain circuit or even severe neurodegenerative disorders [77]. The data in clinical patient populations suggest that MMPs may disrupt the permeability of BBB and interfere with cell signaling in the neurovascular unit. Thus, the validation of MMP blockers serve as a therapeutic opportunity, because BBB perturbations may also occur in neurodegeneration. Hence, MMPs and associated mechanisms may also be potential targets for neurodegenerative disorders [79].

## 4. Involvement of MMPs in NDs

### 4.1. Parkinson’s Disease (PD)

With multifaceted etiologies and about two percent prevalence in the older populations (mainly >60 age), PD is one of the most common, long-term, age-related ND, which has been causing significant burden and disability in the quality of life [80]. It is proposed that the progression and pathogenesis of PD is associated with prominent characteristic i.e., α-synuclein inclusions in the Lewy bodies in affected areas of brain [81]. Such inclusions are made up of parkin, fibrillar, synphilin, α-synuclein, neurofilaments, and proteins of the synaptic vesicle. Being characterized by typical motor symptoms, such as resting tremor, bradykinesia, rigidity, and postural instability, the PD results not only from progressive DAergic damage and neuron deficit in the substantia nigra (SN), rather most probably caused by environmental and genetic factors.

The SN, along with globus pallidus, subthalamic nucleus, and the striatum, modulates motor brain activity [82]. Several symptoms, such as olfactory functional loss, depression, mild motor abnormalities, cognitive and autonomic dysfunction, or rapid eye movement, may antedate the initial clear motor symptoms through a number of years; yet, no one of the either pre-motor symptoms is definite for PD development. Therefore, several hypotheses suggest neuroinflammation to be a pivotal part that is involved in the disease aggravation and promotion [83,84,85].

Various studies on in vivo imaging and postmortem PD tissue revealed microglia overactivation, astrogliosis, and peripheral immune cells infiltration into regions of brain affected in PD [86,87,88], wherein active microglia is detected in certain regions along with Lewy bodies [89]. In concordance with the findings, active microglia have been observed in the SN of familial PD patients after exposure to 1-methyl-4-phenyl-1,2,3,6- tetrahydropyridine (MPTP) [89,90]. The study proposed the early activation of microglia after injecting MPTP, which leads to cell death of neurons, T cell infiltration, and astrogliosis [83]. Activated microglia secrete inflammatory mediators, which thereby increase the levels of proinflammatory cytokine in the CSF and SN in PD [91,92].

Moreover, IL-6 and IL-1β were observed to be higher in CSF of PD patients [93,94]. Some studies proposed that persistent microglial over-activation and proinflammatory cytokines production can contribute to the degeneration of neurons in PD [91,95]. Indeed, damaged DAergic neurons can further activate microglia via the release of neuromelanin and α-synuclein, leading to ROS production [96]. Being secreted by neurotoxin-stressed DAergic neurons, MMP-3 is considered to be an independent player in microglial activation when any other inflammatory molecule lacks, which suggests its vital role in apoptosis. MMP-3 can break the connections between ECM and apoptotic cells by facilitating phagocytosis. Furthermore, it might also activate microglia, contributing to cytokine release and phagocytosis [97]. As per the in vitro study, the induction of extracellular-signal-regulated kinase (ERK) signaling pathway in microglia were observed preceding the stimulation of MMP-3. In addition, hypothesis proposed that both MMP-3 (active and catalytically active) could lead to microglial activation, which thereby exacerbate the apoptosis of deteriorated neuronal cells in order to induce the death of neighboring DAergic neurons. The study is further encouraged by studies that were conducted on postmortem brain, which suggests the progressive DAergic damage of neurons in MPTP treated monkeys and humans for 10 years [88,98]. Besides, microglia activated by MMP-3 could generate super oxides, which is known to be part of in vitro and in vivo DAergic neuronal cell death [99,100,101].

Another study described the active production of MMP-3 by neurons [102]. The upregulation of MMP-9 activity was observed in both SN and striatum following treatment with MPTP and MMP inhibitors protected against neurotoxicity [103]. Furthermore, the localization of MMP-2 and MMP-9 was proposed to be in microglia, and astrocytes and neurons respectively. It was observed that TIMP-2 levels remained unchanged in the same study, but the up surging of TIMP-1 was found in SN not in the hippocampus and cortex [104]. An elevated expression of MMP-9 in SN was further supported by another study, which also suggested the expression of MMP-9 in striatum [105]. Additionally, MMP-9 is widely expressed in astrocytes and microglia, identifying it to be an important factor for neuroinflammatory processes in PD. The experimental studies performed in MMP-9-deficient mice propose that reactive microglia decline the neuronal survival in PD [105]. An elevation in astrocytes and striatal neurons labeled by MMP-9 was also seen in the primate PD model (MPTP-injected macaques). Leakage in BBB was demonstrated in animal PD models in areas of brain and was linked with DAergic neurodegeneration and microglial activation [106,107]. Recently, evidence also suggests the MMPs proteolytic activity, which might be included in the modification of α-synuclein conformation, thereby encouraging its aggregation, microglial activation, and Lewy body formation [108]. It was observed that α-synuclein proteolysis that is dependent on MMP in DAergic neuronal cell line results in an increased formation of aggregates. In this mechanism, MMP-3 was found to be effective, but MMP-1, -2, and -14 exhibited alike features [109]. It was further studied that the cleavage of α-synuclein by MMP showed that both MMP-3 and -1 regulate the increased aggregation of α-synuclein as compared to proteinase K and trypsin [110].

### 4.2. Alzheimer’s Disease (AD)

It is the most common ND, whose well-known features include reduced arborization of dendrites in the subcortical areas and cerebral cortex, and brain atrophy, caused by cell death of neurons [111,112,113]. The key players that are detected in AD are neurofibrillary tangles and the presence of amyloid plaques that are further associated with the atrophy of cerebrum [114]. Amyloid plaques in the parenchyma of brain act as extracellular deposits, comprising of Aβ fibrils [115]. Being considered as a signature lesion for AD, Aβ deposition mainly occurs due to the excessive multiplication of amyloid precursor protein (APP) [116,117]. Certain MP family members, including the ADAM proteins; ADAM-17, -9, and -10, can lead to the cleavage of APP at the cleaving site of α-secretase [118]. Aβ depositions are mainly surrounded by reactive astrocytes, dystrophic neurites, and activated microglia, creating dense core plaques in the regions of brain parenchyma [119].

Various authors pinpoint the role of disturbed ROS generation and antioxidant activity in AD [120]. The fact behind the same is the excessive generation of free radicals, which could act as a driving force for neurodegeneration, along with various stressors, including inflammation, aging, cerebral hypoperfusion, and hypoxia [121]. Some of the theories suggest a fundamental role for reactive microglia close to the amyloid plaques in the CNS. The idea is that reactive microglia produce large amounts of inflammatory chemokines and cytokines, which withstand a prolonged inflammation, eventually leading to neuronal cell death [122]. Besides the well-known effect of neurotoxicity, Aβ can exhibit indirect proinflammatory activity via the microglial activation, which lead to the secretion of TNFα, NO, and super oxides [123,124,125].

Bjerke et al. suggested TIMP-1 and MMP-9 as AD biomarkers, next to P-tau, T-tau, white matter lesions, and Aβ_1–42_, in order to outlook the association between AD and MMPs [126,127]. Amazingly, a relationship between MMP-9 and cognitive impairment was speculated in mild cognitive impairment (MCI) patients [128]. In concordance, Lorenzl et al. speculated elevated MMP-9 levels in AD patients (serum) [129]. The expression of MMP-9 have been observed to be upregulated in patients of AD in neurofibrillary tangles, neuronal cytoplasm, vascular tissue, and amyloid plaques [130]. Yan et al. also exhibited that MMP-9 can degrade amyloid plaques and in vitro Aβ fibrils from APP/PS1 mice in brain slices [131]. An increase in the activity of MMP-9 has been observed in hippocampus while using intracerebroventricular (icv) injections of distinctive Aβ peptides in animal models, which augments cognitive impairment that is induced by Aβ and confirms the results using MMP9 knockout mice and MMP inhibitors [132]. MT1-MMP and MMP-2 expression were found in active astrocytes near amyloid plaques in the transgenic AD mouse model wherein elevated levels of Aβ1–42 augments the formation of MMP-3, -12, and -13 in microglia [133,134]. In addition, MMP-12 aggravates the proteolytic processes by consequent MMP activation, such as MMP-3 and -2 [134].

Furthermore, an increase in the activity of MMP-9 and -2 was observed, and broad-spectrum inhibition of MMP altered the disruption of BBB that is induced by Aβ. Besides, they established these results in transgenic model of mouse by demonstrating the improved MMP-9 immunoreactivity near cerebral capillaries, resulting in the modification of tight junction components. The most important threat for the development of late AD onset is the occurrence of apolipoprotein E ε4 allele in the genome [135]. APOE ε4 leads to breakdown of BBB through activation of cyclophilin A/MMP-9 pathway in the pericytes in both humans and in transgenic mice, which forms guardians of BBB integrity and crucial components of the neurovascular unit. This ultimately leads to the deterioration of the proteins of basement membrane and BBB tight junctions [136,137]. Significant upregulation of MMP-3 levels was observed in plasma, similarly to what was seen in cerebrospinal fluid (CSF) of AD patients [138].

Strikingly, we reported that Aβ oligomers (icv injection) persuades the integrity loss at the blood-cerebrospinal fluid barrier (BCSFB), which is associated with the augmented expression of MMP-3. Besides, the leakage that is induced by Aβ_1–42_ oligomers of the BCSFB could be prohibited by the inhibition of MMP [52]. Leake et al. observed a prominent enhancement in MMP-1 levels in CNS of AD patients [139]. A study conducted by Langenfurth et al. reported the upregulation in the expression of macrophage or microglia in AD patients’ tissues and in a mouse AD model [140]. Lastly, C-reactive protein and TIMP-1 levels were augmented AD patients, and they declined amazingly after acetylcholinesterase inhibitor therapy, which is among the limited existing treatment of AD [141]. Recently, MT5-MMP has been identified as a key player in AD whose colocalization was found to be with amyloid plaques in the brain of AD patients, suggesting it as a participant of the remodeling of injured regions. Moreover, independent efforts from different teams have presented that MT5-MMP mediated APP processing results in the production of fragments via the activity of η-secretase, ultimately resulting in neurotoxic effects in vivo and in vitro [1,142].

### 4.3. Amyotrophic Lateral Sclerosis (ALS)

Amyotrophic lateral sclerosis (ALS) is marked by motor neuronal degeneration in the spinal cord, brainstem, and brain. It involves all of the nerve cells, which affect voluntary muscles, wherein the muscle weakens, leading to atrophy, followed by paralysis, and finally respiratory collapse and death [56]. Remarkably, 1/3 of ALS patients displays pathology or symptoms similar to those of AD [143]. The disease occurrence is comparatively rare, and the incidence is mainly in between the 45–65-year age group of people [144]. Some sporadic and familial cases are mainly due to gene mutation for Zn^2+^-Cu ^2+^ superoxide dismutase 1 (SOD1) [145].

In addition, ALS is related with inclusions of protein, which is composed particularly of cytoplasmic trans active response DNA binding protein 43 (TDP-43) in the damaged spinal cord and brain areas [146,147]. The etiology of ALS is not well-known, but several mechanisms have been suggested, which include oxidative stress damage, glutamate excitotoxicity, neuroinflammation, mitochondrial dysfunction, deficits in neurotrophic factors, and protein misfolding and aggregation [148,149].

Additionally, reactive microglia were shown in the regions of brain in ALS, such as pons, motor cortex, and thalamus. Remarkably, microglia activation was correlated with ALS progression [150]. An in vitro study described that the overexpression of TDP43 by microglia enhanced the production of proinflammatory cytokines upon treatment with LPS in contrast to microglia (wild type) [151]. The hypothesis also proposes that BSCB and BBB breakdown could lead to motor neuronal cell deterioration, because of the significance of BBB in homeostasis regulation in the brain. The involvement of MMPs came from studies on spinal cord and neocortex of ALS patients, considering them as the key players in alteration of barrier, where the localization of MMP-9 was observed in pyramidal neurons in the motor neuron and cortex. Moreover, the activity of MMP-9 was augmented in spinal cord whereas activity of MMP-2 was declined in motor cortex [152]. Because the disruption of BSCB [153,154] in ALS is followed by mRNA downregulation for proteins of tight junction, Miyazaki et al. proposed the involvement of MMP-9 in disruption of barrier [155,156]. The other group speculated diminished activity of MMP-9 during the progression of disease, with the peak ALS onset, and demonstrated the same profiling for MMP-2 [157]. Another two groups suggested significant elevation in active-MMP-9 and pro-MMP-9 of ALS patients’ serum in comparison to healthy individuals [158,159].

It has been reported that, in mild ALS cases, MT-MMP-1, -2, -9, and TIMP-1 expressions are increased in the serum relative to CSF, where MMP-2, MT-MMP-1, and TIMP-1 were unchanged, while the levels of MMP-9 have been declined [160]. Moreover, it was found that MMP-9 increases in CSF of ALS patients, which is quickly progressing; thereby this finding is proposed to be associated with poor patients’ survival, disease progression, and neuronal degeneration. Nonetheless, MMP-2 levels have been progressively declined with the ALS development [161]. In one study, the declining function of MMP-9 by pharmacological, viral, or genetic involvement was speculated for the prolonged survival in a mouse model [162,163,164]. In addition, the pre-expression of MMP-9 only occurred in fast motor neurons that have been shown to be mainly vulnerable to neuronal degeneration in ALS patients. Such outcomes suggest MMP-9 to be a key player in the disease onset and pinpoint it as a therapeutic strategy. Kaplan et al. speculated the early diseased state and MMP-9 expression by neurons. On the other hand, Kiaei et al. focused on later diseased stages and found MMP-9 expression by active microglia, contributing to the theory that microglia-secreted cytokines regulate its pathology [165].

### 4.4. Multiple Sclerosis (MS)

MS is an inflammatory, chronic, and autoimmune CNS disease. The key characteristic of the disease is moderate axonal preservation with demyelinated areas. In comparison with most NDs that are predominant in aged persons, the prevalence of MS in individuals is between 20–45 years of age [166]. The environmental and genetic factors encourage its development, yet the cause is not known. Remarkably, various epidemiological studies showed an association with UVB radiation exposure, smoking, and unsaturated fatty acids intake [167]. MS exist in four main classes, as follows: (1) relapsing-remitting MS (RRMS)—disease interchanges between improvement periods (remission) and deterioration periods (relapses); (2) secondary progressive MS (SPMS)—characterized by constant deterioration of the symptoms; (3) primary progressive MS (PPMS)—shows continuous disease worsening with no relapses or remissions; and (4) progressive relapsing MS (PRMS—rarest category with occasional occurrence of relapses without remission. RRMS is categorized as a neuroinflammatory state at late onset [168]. Various patients go in SPMS after 10 years, which is seen more as neurodegenerative state leading to permanent debility [169]. Besides, inflammation is primarily involved in MS; recent acknowledgements also consider it as a neurodegenerative disorder due to the recent findings [170]. Some of the reports have observed patients suffering from ALS and MS simultaneously [171]. The disrupted BBB in MS leads to the infiltration of peripheral blood leukocyte, succeeded by myelin degradation, and the disruption of axons and cell loss of neurons. Finally, the involvement of MMPs in the above processes is shown by various studies and data [172,173,174]. Alterations in the BBB functionality were identified in the postmortem brains of MS patients [175].

In addition, data suggest the breakdown of BBB further lead to immune cells infiltration [176]. In MS, the secretion of MMPs by several immune and brain cells have been observed, contributing and leading to the breakdown of BBB [177,178]. It has been speculated that the secretion of MMP-9 and -7 occurs in blood vessels of postmortem brain samples and macrophages, respectively [179]. The other study confirmed and showed MMP3 expression in endothelial cells, MMP-1, -2, -3, and -9 in macrophages around necrotic and active lesions [85]. It was examined that CSF samples from PPMS and RRMS patients showed an upsurge in the level of MMP-9 during both phases of MS. Although, MMP-9 was augmented in about ½ of the samples in PPMS patients with smaller amounts than in the remitting-relapsing period. They debated that this pinpoints the fact that macrophages and T-cells are mainly responsible for MMP-9 secretion in MS. Moreover, they suggested the continuous increase in MMP-9, which might lead to the damage of neighboring tissue and cell loss of neurons [180]. Increased MMP-9 levels have also been seen in MS patients’ serum, along with increased TIMP-1 and -2. The same study [181] highlighted the correlation of these augmentation with the lesions that were detected by MRI. Nevertheless, the study found an increase in the MMP-9 levels in serum with no such TIMP-1 elevation.

Other data developed a comparison of the levels of MMP-1, -3, -7, -9, and -14, and TIMP-1 in MS patients’ blood, finding that all were upregulated with the exception of MMP-14 [182]. In an interesting study, transgenic mice using the EAE model express TIMP-1, which had a general phenotype, yet symptoms of experimental autoimmune encephalomyelitis (EAE) were reduced [183]. Remarkably, some studies, using (EAE) model, proposed a limited amelioration and restoration of BBB after the administration of MMP inhibitors [184]. Finally, MMP-9 knockout mice have been less susceptible to EAE induction [185].

Apart from the leakage, the activation of BBB also takes place, which means that cells constituting the BBB, together with pericytes, astrocytes, and endothelial cells, initiates the expression and secretion of several factors that are part of the functioning and recruitment of leukocytes [175]. The continuous migration of leukocyte mainly occurs via BBB in active lesions in MS, which is rigorously regulated by various molecules, including chemokines, integrins, cell adhesion molecules (CAM), and cytokines.

Further, the infiltration of leukocytes exacerbates the breakdown of BBB, as shown in in vitro studies [186]. An interferon β treatment downregulated the MMP-9 expression and abolished the MMP-2 expression, therefore reducing consequent T-cell migration [187]. Newman et al. presented MMPs microinjection into white matter, which results in axonal injury. The most potent MMP has been found to be MMP-9 out of the several MMPs, succeeded by MMP-7 and MMP-2 [188]. The suggested mechanism of action of MMP is via ECM degradation, yet MMPs have a well-developed function in the apoptosis of distinctive cell types [189]. Some favorable roles of MMPs have also been observed in MS [190]. For example, MMP-9 has a distinguishing role in the process growth of oligodendrocyte [152]. It has been considered that this can be the reason of diminished remyelination and a reduced number of oligodendrocytes in MMP9/-12 null mice and MMP-9 [191].

**Figure 2:**
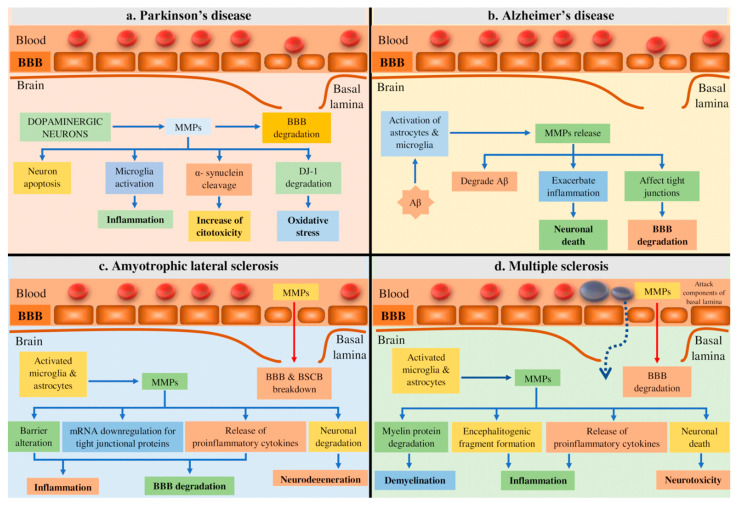
Highlighting the role of MMPs in the pathogenesis of neurodegenerative diseases. (**a**) MMPs in PD contributes to microglia activation, dopaminergic apoptosis, DJ-1 degradation, and α-synuclein cleavage; (**b**) in AD, the deposition of amyloid plaques results in the activation of astrocytes and microglia, inducing the MMP production, which contribute to the BBB degradation; (**c**) in ALS, MMPs contributes to BBB alteration, the downregulation of tight junctional proteins, and neuronal degradation; and, (**d**) MMPs contribute to MS pathogenesis via BBB degradation, myelin degradation, proinflammatory cytokine release, and infiltration of immune cells. Legends Aβ, β-amyloid; AD, Alzheimer’s disease; ALS, Amyotrophic lateral sclerosis; BBB, blood brain barrier; BSCB, blood-spinal cord barrier, DJ-1, protein in humans encoded by PARK7 gene; MMP, matrix metalloproteinase; MS, Multiple sclerosis; PD, Parkinson’s disease.

Figure 2 highlights the role of MMPs in the pathogenesis of NDs.

### 4.5. Huntington’s Disease (HD) and Other NDs

The involvement of MMPs has been observed in other NDs, including HD. HD is an autosomal dominant, inherited ND, which is associated with chromosome 4 mutation in huntingtin (Htt), a protein that is responsible for gene coding. The disease is characterized by a reduction in the mental ability and muscle coordination. It has been observed that Htt (mutant) proteolysis contributes to its pathology, yet the role of Htt is not well defined [192]. Apart from the role that is played by calpains and caspases as proteases in HD, it is speculated that MMPs play a distinguishing role in Htt cleavage. It was observed that the knock down of MMP-10, -14, and -23 in striatal cells (cultured) expressing mutant Htt declines the toxicity. In addition, MMP-10 is involved in the direct cleavage of Htt, and the generation of toxic fragments of Htt is diminished upon MMP-10 silencing [193]. HD patients’ analysis suggested an upsurge of MMP-9 in contrast to controls, together with cytokine upregulation in cerebellum and cortex [194]. The major areas affected in HD is the striatal pathway, wherein the upregulation of IL-10 and chemokine ligand 2 (CCL2) takes place. New data for MMP-9 involvement in HD mainly come from the 3-nitropropionic acid animal model [195]. The group of authors exhibited MMP-9 to be responsible for the disruption of BBB that takes place in HD. Furthermore, a significant elevation in the MMP-9 levels were seen in the plasma of HD patients and R6/2 mouse model of HD [196]. It has been suggested that MMP-9 (together with VEGF, IL-6, and TGF-β) serves as a HD biomarker. As long as the involvement of TIMP is concerned, it has been speculated that levels of TIMP-1 and -2 augments in the CSF of HD patients [197].

Different MMPs were shown to be altered in individuals suffering from dementias in other NDs. In people with frontotemporal dementia, declined TIMP-2 levels were observed in the serum, and the downregulation of TIMP-1 was indicated in people with vascular dementia [128]. Augmentation in the levels of active MMP-2, proMMP-9, TIMP-1, and TIMP-2 were shown in a rare type of dementia, Creuztfeldt–Jakob disease [198].

Table 1 summarizes the role of MMPs in NDs.

## 5. Potential Role of MMP-3 in Neurodegeneration

In the last few years, there has been wide attention on the role of MMP-3 in several mechanisms taking place in the brains of mammals in biological as well as pathological conditions [8]. Numerous studies have shown the involvement of MMP-3 in neurodegeneration. Although neurodegeneration is not well understood process, but neuroinflammation and neuronal apoptosis are considered to function. In vivo, MMP-3 may contribute to neurodegeneration that is based on available data by participating in these processes. The extracellular activation of proMMP-3 is performed by the serine proteinases [208]. MMP -3 cleaves the components of ECM, such as aggrecan, fibronectin, laminin, tenascins, as well as TNF-α and interleukin 1b [209]. Choi et al. showed the proMMP-3 activation also occuring inside the DAergic neurons, which go through cellular stress, where serine proteinases have activated pro-MMP-3 zymogen [102]. MMP-3 plays a pivotal function as a signaling molecule in apoptosis somewhere downstream and upstream of caspase 12 and caspase 3, respectively, under stressful conditions.

**Figure 3:**
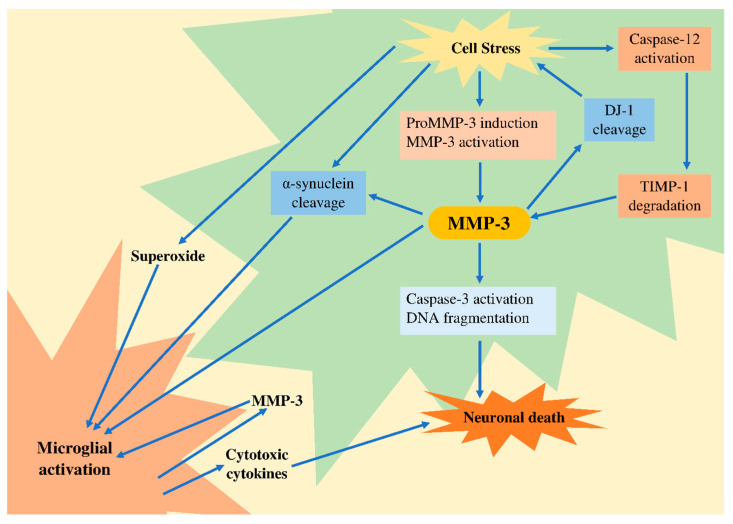
Possible role of MMP-3 in neurodegeneration. DJ-1, protein in humans encoded by PARK7 gene; DNA, deoxyribonucleic acid; MMP, matrix metalloproteinase; TIMP-1, tissue inhibitor of metalloproteinase.

The elevated activity of MMP-3 is due to the proteolytic activation of the zymogen, induction of gene expression, and degradation of TIMP-1. Moreover, active MMP-3 is extracellularly released, leading to the activation of microglia, which then produces various cytotoxic proinflammatory molecules. These harmful molecules thereby prompt the neuronal death via the activation of death receptors, leading to oxidative stress. Additionally, the production of MMP-3 by activated microglia occurs in the ECM, thereby accelerating the neuroinflammation (Figure 3). By triggering various pathways, MMP-3 may be required for organizing rapid and effective clearance and death of the neurons. An increased MMP-3 has been shown in different PD experimental models.

Various data support the potential role of MMP-3 in NDs. In an animal PD model, a increase in MMP-3 immunoreactivity was observed in the SN region, such as animals (rats) that are injected with the selective DAergic toxin; LPS and 6-OHDA [109,210]. In another MPTP induced animal PD model, the DAergic neuronal degeneration in the SN was lesser in MMP-3 KO animals relative to wild type [211]. Cell culture models of PD that are produced by tetrahydrobiopterin and MPP+ exposure also exposed the MMP-3 induction [99,210]. The neuronal cell demise in aforementioned PD models was diminished by gene knockdown and pharmacologically inhibition method [102]. MMP-3 is a driving factor in prompting neuroinflammation in response to oxidative or stress to neuronal cells; thereby, neuroinflammation is the central process towards the neurodegeneration. Because the release and production of MMP-3 occurs from these neurons, the SN could be more susceptible to neuroinflammation and lastly neurodegeneration. Moreover, MMP-3 contributes the disruption of BBB that might permit immune cell infiltration to the damaged areas [212].

The published study also implies a role of MMP-3 in the AD pathophysiology. It has been proposed that that toxicity of Aβ may elicit the induction of MMP-3 activity and expression [49]. Mixed hippocampal neuronal culture and astrocytes that were treated with Aβ_1–40_ express MMP-3 together with increased catalytic activity of MMP-3 [213]. Aβ_1–42_ induces the expression of MMP-3 in microglia [130]. Moreover, the protein level of MMP-3 is augmented in the AD brains and it has been identified in the astrocytes of white matter and interstitium between myelinated axons in AD patients [214]. Figure 3 depicts the role of MMP-3 in neurodegeneration.

## 6. Therapeutic Opportunities

Over the last decades, the role of MMPs became well appreciated in NDs. Several MMPs have been involved in the development and progression of NDs, thereby opening up the possibility of therapeutically targeted MMPs. Neuroinflammation is seen either during or before the development of the pathological features of NDs. MMPs increase the BBB permeability during neuroinflammation by the destruction of the tight junctional proteins or degradation of the ECM, thereby leading to immune cell infiltration via BBB and cell demise [211].

The inhibition of MMP activity in neurodegenerative disorders occurs at different phases of disease progression. An inflammatory stimulus (e.g., burns, protein aggregates, or infection) triggers the expression of MMP, in turn inducing an inflammatory process, therefore opening up the possibility of anti-inflammatory drugs as a therapeutic agent in abolishing the MMP activation or expression. Consequently, the use of synthetic broad-spectrum inhibitors might target and inhibit MMPs. Yet, the specific inhibition of MMP could be suitable, which might prevent the undesirable effects of broad-spectrum MMP inhibitors. Ordinarily, the inhibition of MMP occurs by binding to the Zn^2+^ in the active site [215,216]. Moreover, interference with the substrates that are involved in the sequence of MMPs could also serve as a potential target or have therapeutic value.

### 6.1. Alzheimer’s Disease

The MMP inhibition in AD is mainly dependent on the seemingly favorable effect of MMP-9 and it is very speculative. This is because of the involvement of MMP-9 in degradation of amyloid plaques and contribution to the Aβ clearance from the brain. Moreover, MMP-2 has been reported in the cleavage of Aβ at the α-secretase [164,217,218]. It was also speculated that full-length APP is also cleaved by MMP-2, which suggests that it can either degrade Aβ in the ECM or generate α-APPs at the plasma membrane that can result in a decline of Aβ burden in the CNS [219].

Several data pinpointed the role of MMP-9 and -2. Similarly, treatment with GM6001, a broad spectrum MMP inhibitor, caused an upsurge in Aβ in transgenic mice overexpressing the Swedish variant of APP [220]. In an in vitro study, GM6001 has been observed to inhibit the alterations that are induced by Aβ in BBB permeability and ZO-1 expression in an in vitro study. Likewise, GM6001 prevents the degradation of blood-CSF barrier induced by Aβ oligomer [52]. Furthermore, GM6001 induced MMP inhibition diminished the oxidative stress that is linked with CAA in a transgenic mouse model of AD [221,222,223]. TIMPs have been found near the neurofibrillary tangles and Aβ plaques of brain samples that are affected by AD. TIMPs and MMPs were found to encourage the lesions’ evolution. In addition, MMPs are well-known to be produced in large amounts at the sites of lesions by immune cells of effected areas, and TIMPs might regulate the MMP activity, indicating that TIMPs’ deregulation also results in AD progression [223,224]. The importance of MMPs in AD is not well established [225,226,227]. 

### 6.2. Parkinson’s Disease

In PD, as long as therapeutic strategies of MMP inhibition are understood, the expression MMP-1, -2, -9, and TIMP-1 and -2 have been reported in the SN of postmortem brain samples of PD patients [104]. Thus, MMP inhibition could hold promise for PD management, due to DAergic neuronal death, which has been found to be linked with MMP release. The apoptosis of DAergic neurons leads to the release of MMP-3 that contribute to the process of microglial activation in vitro, also suggesting MMP-3 as a signaling molecule. The proinflammatory cytokines are released by activated microglia that could cause the cell death of neurons [97]. In mouse mesencephalic cells, the treatment of a selective dopaminergic neuronal toxin, tetrahydrobiopterin (BH4), diminished the survival of cell. However, the treatment of cells with MMP-3 inhibitor, N-isobutyl-N-[4-methoxy phenylsulfonyl]-glycyl hydroxamic acid (NNGH), extended cell survival through the decline of TNF-α secretion from activated microglia [102].

### 6.3. Amyotrophic Lateral Sclerosis

A number of theories were suggested concerning the role of MMPs in the ALS development. Moreover, the specific inhibition of MMP could be therapeutic target in ALS. In a study, the MMP-9 expression and immunoreactivity were increased in G93A SOD1 mice (spinal cord tissue), a familial ALS model by crossing MMP-9 knockout mice with G93A SOD1 mice [165]. The diminished activity of MMP-9 has been shown to extend survival in the mutant SOD1 expressed mouse model of ALS, indicating MMP-9 to be better therapeutic target [224]. In general, neuronal TNF-α is stimulated by MMP-9 by cleaving from its membrane-bound form, prompting the neuronal cell demise via the activation of other proinflammatory cytokines [165]. Unusually, degraded matrix elements and elevated MMP-9 levels encourage the progression of ALS [152].

### 6.4. Multiple Sclerosis

Numerous reports on the utilization of synthetic MMP inhibitors have been developed to improve the EAE, and protease inhibitor therapy were recommended in EAE in early 1982 [228]. The activity of MMP has been suggested to upsurge three times in the CSF of two acute EAE models [229]. MMP inhibitors, which are broad spectrum in nature, such as RO31-9790, GM6001, UK221,316, BB1101, and d-penicillamine were indicated to be advantageous in EAE [230,231,232,233]. The levels of MMP-9 were elevated in the CSF and at the lesion sites of MS. Likewise, correlation of MMP-9 with the disrupted BBB was also seen in MRI reports [234]. After the clinical disease onset, GM6001 administration hindered with EAE development, and also diminished the clinical symptoms in SJL/J mice. Similarly, decrease in the activity of MMP-9 was shown in treated mice [234]. It has been considered that the inhibition of MMP leads to the repair of disrupted BBB, therefore ameliorating the inflammation.

Another study showed a reduction in the clinical signs in the MS patients while using RO31-9790 in EAE model three days after or on the day of disease induction [235]. The other broad-spectrum inhibitor, BB1101, ameliorated symptoms in SJL/J mice and declined the intensity of disease in Lewis rats [229,236]. BB1101 therapy decreased the demyelination and glial scar, which was also efficient in the prolonged relapsing in SJL/J mice in EAE. Besides, B1101 altered the profile of cytokines to an anti-inflammatory state [184]. To date, no such molecular mechanism has been known to delineate the amelioration of symptoms in MS, but broad-spectrum MMP inhibitors have been proposed to inhibit the migration of immune cells into the brain, thereby leading to decreased TNF levels and diminished demyelination through ADAM17 inhibition [237,238].

## 7. Conclusions and Future Directions

MMPs play a pivotal, yet multifaceted, role in NDs by several cell signaling and functions. They degrade ECM and disrupt the BBB tight junctions, thereby, they act as driving forces in the progression of NDs. MMPs, along with their inhibitors, TIMPs, mediate functions of cell signaling, which are vital in numerous diseases. In this article, we have reviewed the multifaceted functions of MMPs in main NDs, mainly PD, AD, ALS, MD, and HD, as well as their potential therapeutic interest. Although the etiology and potential causes of NDs remain widely indefinable, MMPs are evidently involved in the progression of these diseases.

New emerging evidence demonstrated the effects of MMPs in NDs, including microglial activation, amyloid peptide degradation in AD, apoptosis of daergic neurons in PD, damage to white matter in VCI patients, and disruption of BBB in MS and ALS [239]. Moreover, in the aforementioned diseases, the key role of the neuroinflammatory response that is regulated by production of MMP has also been discussed. Numerous MMPs are associated with NDs, including MMP-2, -3, and -9, as the pivotal players in the diseases that are discussed in this review. They act via common route of pathological alterations in the homeostasis of CNS, resulting in increased CNS permeability, thereby leading to cell demise. Because the MMPs’ involvement in the regulation of the pathological modifications in NDs is unraveling, there is a need to explore these in the clinics. The animal studies showed that MMP inhibition can decrease the tissue injury and damaged vessels in each of these diseases. Over time, several agents have been developed in order to overcome challenge and open up the possibilities of making selective modulators of MMPs to decipher the multifaceted functions of MMPs. The multifaceted role of MMPs impedes the attempts of broad spectrum MMP inhibitors as strategic target. However, thefine-tuning between MMPs and TIMPS is probably a key to the development of efficient and selective therapeutics whose investigation needs to be continued.

Future directions could include, but are not limited to, the use of humanized self-organized models, organoids, 3D cultures, and human micro vessel-on-a-chip platforms, especially those that are amenable for advanced imaging, since they enable real-time monitoring of BBB penetration and permeability [240,241,242].

## Figures and Tables

**Table 1:** The role of MMPs in neurodegenerative diseases.

MMPs Involved/ Neurodegenerative Disease	Model System	Role of MMPs	Ref.
MMP-2/PD	In vitro (PC12 cells)	Activates microglia	[97]
	Patients	Detected in microglia and astrocytes	[99]
	In vitro (neuron-glia culture)	Induces DAergic neuronal death in culture of glia-neuron (mesencephalic)	[103]
MMP-2/AD	In vivo (rats)	Involved in synaptic plasticity	[199]
	In-vitro (microglial cell line)	Increased microglial expression after Aβ oligomer stimulation	[133]
MMP-1/AD	In vitro (primary astrocytes)	Low MMP-9 levels and decreased MMP-2 activity after Aβ oligomer stimulation.	[139]
	Patients	Increased MMP-1 levels in AD patients	[200,201]
MMP-3/PD	In vitro (primary cultured DAergic neurons)	MMP-3 neuronal secretion	[102]
	In vitro (primary mesencephalic cultures)	Induces NO production in microglia	[98,100]
	In vitro (human DAergic neuroblastoma)	α-synuclein proteolysis	[202]
MMP-3/AD	Patients	Significant upregulation of MMP-3 plasma levels	[138]
	In vivo (icv injections of Aβ oligomer)	Increased MMP-3 expression	[52]
	In vitro (APP-CHO cells)	Ability to degrade Aβ	[203]
	In vivo (icv injection of Aβ oligomer)	Enhanced permeability of BCSFB	[52]
MMP-2/ALS	Patients	Increased permeability of BBB	[204]
	Patients (serum)	To evaluate ALS progression	[161]
MMP-9/AD	In vitro (astrocytes)	Detected in astrocytes when treated with fibrillar and soluble Aβ	[205]
	In vivo (rats)	Involved in synaptic plasticity	[199]
	In vivo (mice)	Increased levels in hippocampus on icv injection	[132]
	Patients (CSF)	Activation of MMP-9/CypA in pericytes, BBB disruption	[137]
	In vitro (isolates from brain of patients)	Cleavage of Aβ_1-40_ by MMP-9	[206]
MMP-3/ALS	In vivo (G93A SOD1 mice)	Upregulation of neuronal FasL and TNF	[157]
	In vivo (mutant SOD1 transgenic mice)	Dysregulated MMP-3 activity with ALS progression	[165]
	In vivo (G93A SOD1 mice)	Encourages motor cell death in neurons	[165]
MMP-1/MS	Patients (monocytes)	Increased mRNA levels of MMP-1	[182]
	Patients (postmortem brain samples)	Weak astrocytic expression	[207]
MMP-3/MS	Patients (monocytes)	Increased mRNA levels of MMP-3	[182]
	Patients (postmortem brain samples)	Expression in endothelial cells	[207]
MMP-9/HD	In vivo (3-nitropropionic acid animal disease model)	Increased expression of MMP-9	[195]
MMP-9/PD	Patients (postmortem brain tissues)	Increased expression of MMP-9 in SN	[116]
	In vivo (MPTP induced PD in monkey and mouse model)	Primary localization of MMP-9 in neurons	[103]
MMP-7/MS	Patients (monocytes)	Increased mRNA levels of MMP-7	[182]
	Patients (postmortem brain samples)	Secreted by blood vessels	[179]
MMP-9/ALS	Patients (CSF and skin) Patients (CSF)	Elevated in CSF and skin Low CSF levels of MMP-9	[160,161]
	Patients (serum)	MMP-9 as marker distinguishing between healthy individuals and ALS	[204]
MMP-10/HD	In vitro (striatal cell culture)	Cleaves huntingtin	[181]
MMP-9/MS	Patients (CSF samples)	Secreted by macrophages and T-cells, leads to damage of tissue	[160]
	Patients (serum)	Increased serum levels together with TIMP-1 and -2	[173]
MMP-14/HD	In vitro (striatal cell culture)	MMP-14 knockdown reduces toxicity	[181]
MMP-12/AD	In vitro (microglial cell line)	Increase in microglia	[126]
MMP-23/HD	In vitro (striatal cell line)	MMP-23 knockdown reduces toxicity	[193]
MMP-13/AD	In vitro (microglial cell line)	Increase in microglia	[126]

Aβ, β-amyloid; AD, Alzheimer’s disease; ALS, amyotrophic lateral sclerosis; APP, amyloid precursor protein; BBB, blood-brain barrier; BCSFB, blood-CSF barrier; CSF, cerebrospinal fluid; Cyp A, cyclophilin A; Dopaminergic, DAergic; FasL, Fas ligand; G93A SOD1 mice, transgenic mice form; HD, Huntington’s disease; i.c.v., intracerebroventricular; MMP, matrix metalloproteinase; MPTP, 1-methyl-4-phenyl-1,2,3,6- tetrahydropyridine; MS, multiple sclerosis; NO, nitric oxide; PC12 cells, classical neuronal model; PD, Parkinson’s disease; SOD, superoxide dismutase; TIMP, tissue inhibitor of metalloproteinases; TNF, tumor necrosis factor.

## Data Availability

Not applicable.
